# Arbovirus Diseases, Southeastern United States

**DOI:** 10.3201/eid1911.130650

**Published:** 2013-11

**Authors:** Lisa M. Gargano, Jeffrey Engel, Gregory C. Gray, Kelly Howell, Timothy F. Jones, Wilbur K. Milhous, J. Glenn Morris, Daniel G. Mead, Carol Rubin, Thomas R. Unnasch, Christopher W. Woods, James M. Hughes

**Affiliations:** Emory University, School of Medicine, Atlanta, Georgia, USA (L.M. Gargano, K. Howell, J.M. Hughes);; Council of State and Territorial Epidemiologists, Atlanta (J. Engel);; University of Florida, Gainesville, Florida, USA (G.C. Gray, J.G. Morris, Jr.);; Tennessee Department of Health, Nashville, Tennessee, USA (T.F. Jones);; University of South Florida, Tampa, Florida, USA (W.K. Milhous, T.R. Unnasch);; University of Georgia, Athens, Georgia, USA (D.G. Mead);; Centers for Disease Control and Prevention, Atlanta (C. Rubin);; Duke University Medical Center, Durham, North Carolina, USA (C.W. Woods)

**Keywords:** arbovirus, southeastern United States, arbovirus diseases, mosquitoes, viruses

Historically, the southeastern United States has experienced repeated epidemics of yellow fever and malaria. Endemic to this region are several arboviruses, including St. Louis encephalitis, West Nile, eastern equine encephalitis, and LaCrosse viruses and the mosquito vectors for several arboviruses, including *Aedes aegypti*, *Ae. albopictus*, and several *Culex* species mosquitoes. 

The ecologic conditions in the southeastern United States seem poised to support endogenous transmission of introduced vector-borne pathogens normally endemic to the tropics, a threat recently highlighted by local transmission of dengue virus in Key West and other areas of Florida (*1*). The long hot summers, long rainy seasons, and periods of drought in the southeastern United States make for optimal breeding conditions for mosquitoes. The busiest airport in the world, Hartsfield-Jackson Airport in Atlanta, is a major entry point for travelers from tropical regions, some of whom take connecting flights to other cities in the Southeast and while others go farther. Infected travelers could result in an increased risk for the introduction of exotic pathogens, including arboviruses, to the region. In addition, bird migratory routes cover much of the Southeast. 

These circumstances emphasize the need for state agencies to exchange surveillance data and lessons learned. To meet this need, the Southeastern Regional Center of Excellence for Emerging Infections and Biodefense convened a conference, Arbovirus Infections in the Southeastern US, on February 11–12, 2013 (www.serceb.org). The objectives were to 1) describe the recent epidemiology of arboviruses endemic to the southeastern United States; 2) identify vectors of concern in the region and discuss the risks for introduction of exotic arboviruses such as dengue, Japanese encephalitis, Rift Valley fever, chikungunya, and yellow fever viruses; 3) discuss challenges to surveillance and the current status of regional public health preparedness and response capacity; and 4) summarize regional perspectives on research needs and priorities for addressing arbovirus diseases. Participants included representatives of state health departments, mosquito control organizations, academic institutions, and federal agencies.

Representatives from 5 southeastern states (Florida, Georgia, North Carolina, Tennessee, and Mississippi) shared their recent experiences. Several issues and challenges were commonly expressed. Arbovirus transmission in the region is focal, sporadic, and unpredictable. State public health officials reported conducting various surveillance activities focused on mosquitoes, birds, sentinel chickens, horses, and humans. There was widespread concern regarding lack of adequate and stable funding for surveillance, particularly mosquito pool surveillance (which is labor intensive), sentinel chicken surveillance, and public education and laboratory support. State representatives believed that improved coordination among organizations (including consistency in approaches to surveillance among states in the region) and improved timely dissemination of information to the public would improve preparedness and response to arbovirus disease outbreaks. Participants expressed concerns regarding the lack of surveillance and response capacity for potentially emerging exotic arboviruses such as chikungunya and Rift Valley fever viruses. Clinicians should be alert to unexplained febrile illness and a suggestive travel or exposure history. It was suggested that detection of Flanders virus in mosquito pools might be an indicator of risk for subsequent emergence of West Nile virus (*2*).

Several environmental issues in the Southeast influence arbovirus transmission. These issues include unique urban ecology in cities like Atlanta because of poor maintenance of residential swimming pools; wastewater treatment facilities in Atlanta and elsewhere have also presented challenges. In addition, research in Atlanta has identified the major role of combined sewer overflow systems as vector habitats, which increase the risk for human exposure to potentially infected mosquitoes (*3*). Other identified concerns were the lack of entomologists and shortages of surveillance and mosquito control personnel in the region.

The participants identified several overarching research priorities. The effects of climate change on arbovirus transmission and vector breeding sites are currently unknown. The Southeast is exceptionally vulnerable to some effects of climate change and severe weather events—including rising sea levels, extreme heat events, and decreased water availability—which can play a role in altering vector habitat and arbovirus transmission (*4*). The need to understand spatial and temporal influences on the heterogeneity of infection rates was discussed. In particular, there is a need to appreciate the variability in ecology across the region and the potential effects of this variability on the design of appropriate interventions. There is also a need for research to assess the effects of mosquito control programs on arbovirus transmission and evaluation of emergency mosquito control measures. The occurrence of hybrid species of potential vectors in the region raises questions about their role in arbovirus transmission and merits further assessment (*5*–*7*). Mathematical modeling can be used to examine predictors of arbovirus transmission and the potential effectiveness of community programs for educating the public about disease prevention.

The group discussed the lack of countermeasures for arbovirus infections. Ongoing efforts to develop a vaccine for dengue were noted. Challenges in evaluating potential therapeutic options for West Nile neuroinvasive disease were acknowledged; these patients are often admitted to smaller hospitals not accustomed to participating in clinical trials. When a promising vaccine candidate becomes available, the unpredictable nature of the annual occurrence of West Nile encephalitis cases will pose a challenge to the design of a phase III vaccine trial (*8*). Availability of point-of-care rapid diagnostic tests could strengthen surveillance programs and encourage timely reporting of disease by clinicians.

Participants discussed the need to strengthen interstate collaboration for a needs assessment to determine what information is already being collected and to identify critical gaps to building more granular datasets that will allow for regional modeling and other analyses. The need to develop research collaborations and convene regional meetings to share results among public health, mosquito control, and academic partners was emphasized, as was the potential value of student organizations that would be able to assist the states in control, research, education, and surveillance of arboviruses (*9*,*10*). Finally, because of the risk for changes in the epidemiology of established arbovirus diseases in the region (*11*), the group identified the need for the following: an inventory of arbovirus surveillance activities, including specific information on laboratory testing; updated technical guidance on surveillance and mosquito control procedures; and dissemination of best practices. The group committed to meet again within a year to assess progress and barriers.
